# Early Right Motor Cortex Response to Happy and Fearful Facial Expressions: A TMS Motor-Evoked Potential Study

**DOI:** 10.3390/brainsci11091203

**Published:** 2021-09-13

**Authors:** Sara Borgomaneri, Francesca Vitale, Simone Battaglia, Alessio Avenanti

**Affiliations:** 1 Centro Studi e Ricerche in Neuroscienze Cognitive, Dipartimento di Psicologia, Campus di Cesena, Alma Mater Studiorum-Università di Bologna, 47521 Cesena, Italy; simone.battaglia@unibo.it; 2 IRCCS Fondazione Santa Lucia, 00179 Rome, Italy; 3 Instituto Universitario de Neurociencia (IUNE), Universidad de La Laguna, 38200 Santa Cruz de Tenerife, Spain; frvitale@ull.edu.es; 4 Centro de Investigación en Neuropsicología y Neurosciencias Cognitivas, Universidad Católica Del Maule, Talca 3460000, Chile

**Keywords:** emotional facial expressions, transcranial magnetic stimulation, motor evoked potentials, early motor reactions, empathic traits

## Abstract

The ability to rapidly process others’ emotional signals is crucial for adaptive social interactions. However, to date it is still unclear how observing emotional facial expressions affects the reactivity of the human motor cortex. To provide insights on this issue, we employed single-pulse transcranial magnetic stimulation (TMS) to investigate corticospinal motor excitability. Healthy participants observed happy, fearful and neutral pictures of facial expressions while receiving TMS over the left or right motor cortex at 150 and 300 ms after picture onset. In the early phase (150 ms), we observed an enhancement of corticospinal excitability for the observation of happy and fearful emotional faces compared to neutral expressions specifically in the right hemisphere. Interindividual differences in the disposition to experience aversive feelings (personal distress) in interpersonal emotional contexts predicted the early increase in corticospinal excitability for emotional faces. No differences in corticospinal excitability were observed at the later time (300 ms) or in the left M1. These findings support the notion that emotion perception primes the body for action and highlights the role of the right hemisphere in implementing a rapid and transient facilitatory response to emotional arousing stimuli, such as emotional facial expressions.

## 1. Introduction

Emotional facial expressions are a gold mine of social information, and the ability to accurately perceive and respond to them is crucial to social success. Humans show remarkable abilities to identify and judge others’ expressions even in difficult and ambiguous conditions, e.g., when faces are presented for less than one tenth of a second [1,2,3,4]. Meta-analyses addressing the neural bases of facial perception, have shown that the processing of emotional faces is associated with increased activation in a number of visual, limbic, temporoparietal and prefrontal areas, as well as in several motor structures [5,6,7]. Modulations of these areas occur rapidly as shown by several electroencephalography studies [8,9], in keeping with the notion that emotional signals rapidly engage neural resources to efficiently process and react to stimuli relevant for survival.

Scholars have commonly proposed that activation in motor areas during perception of emotional faces could reflect either the activation of motor resonance processes—which would support covert/overt mirroring of the observed expression in the observer [10,11,12]—and/or the activation of motor programs to implement adaptive motor responses (e.g., orienting/freezing or fight/flight responses) [13,14,15]. However, because imaging and electrocortical techniques suffer respectively from relatively low temporal and spatial resolution, and both methods can hardly distinguish between excitatory and inhibitory processes, previous works using these approaches were unable to establish the functional meaning of motor activations during observation of the emotional faces.

Transcranial magnetic stimulation (TMS) is a valuable method to investigate the temporal dynamics of the motor system during perception of emotional faces, via stimulation of the primary motor cortex (M1) and the consequent induction of motor-evoked potentials (MEPs) in target muscles. The amplitudes of TMS-induced MEPs provide an instantaneous readout of the excitability of the corticospinal system, allowing to probe distinct motor representations with high temporal resolution, and, importantly, to distinguish between excitatory (MEP increase) and inhibitory (MEP decrease) motor processes.

However, to date, TMS has been mostly used to investigate the involvement of the motor system in processing emotional signals during observation of complex emotional scenes [16,17,18,19,20,21] or emotional body postures [21,22,23,24,25,26,27,28,29], rather than observation of emotional faces (see below). A review of this work is nevertheless useful to understand the relation between emotional signals and motor processes across different classes of stimuli. Studies using complex natural scenes have typically selected stimuli from the international affective picture system (IAPS) and reported increased MEPs when participants were presented with both pleasant and unpleasant scenes [16,17,18]. A few studies using the same set of IAPS stimuli reported higher facilitation for unpleasant scenes, although this effect was probably due to the higher arousal of these scenes [19,20,21]. These facilitatory modulations have been commonly interpreted in terms of increased motor readiness to relevant arousing stimuli, reflecting preparation of adaptive motor responses [13,14,15]. Notably, in most of such studies, motor excitability was tested in a relatively late time window (i.e., at >300 ms after stimulus onset; but see [21]), when the magnitude of brain response to emotional scenes is typically similar for positive and negative stimuli and likely reflects enhanced resource allocation to motivationally relevant cues [30,31,32]. Only one study by Borgomaneri et al. [21], explored an earlier time point (i.e., 150 ms) and found increased excitability in the left M1 for negative scenes associated with higher arousal [21].

One potential issue in these previous studies, is that emotional scenes very often conveyed emotional meanings through the facial expressions of the depicted actors, suggesting that enhanced motor readiness could be triggered by emotional faces alone rather than (or in addition to) emotional contextual cues. Even more critically, emotional scenes often showed not only the faces but also the actors’ dynamic motor actions, whereas neutral scenes typically involved neutral contexts with no humans, raising the possible concern that increased motor excitability for emotional scenes could be due to the effect of observing human actions, i.e., motor resonance (see discussion in [22]), rather than any emotion-related neural modulations.

While studies testing natural scenes have reported increased motor excitability for emotional scenes, research investigating MEP response to the observation of emotional body expressions has commonly reported reduced motor excitability for emotional bodies [23,24,25,26,29]. These studies have used sets of validated pictures showing body postures in isolation with no facial or contextual cues, while participants were asked to actively recognize fearful and happy body postures (with comparable arousal), emotionally neutral dynamic body postures (with implied motion comparable to emotional postures) and neutral static postures. Notably, these studies explored early time windows and reported that seeing emotional bodies reduced motor excitability quite early in time (i.e., at 70–150 ms from picture onset) when the right M1 was targeted [23,24,25,26], with a tendency for fearful bodies to induce the earliest responses [25].

The early suppression of motor output has been interpreted as reflecting an orienting/freezing mechanism supporting the monitoring of relevant emotional signals [23,24,25,26,29]. However, it remains unclear whether early orienting is specific to emotional bodies, as a study using IAPS found no MEP reduction at 150 ms for emotional scenes [21]. At a later point (i.e., at 300 ms), two studies reported motor facilitation for dynamic expressions compared to static body postures, no matter whether the dynamic expressions were emotional or neutral, suggesting that these responses reflected a motor mapping of the observed action (i.e., motor resonance) rather than emotion-related modulations [22,23].

In sum, the literature addressing MEPs during perception of emotional scenes and body postures generically support the notion that the observers’ motor system could reflect both emotion-related adaptive responses to arousing stimuli (increased readiness, or orienting/freezing) and motor resonance. However, does the perception of emotional faces induce similar processes in the observers’ motor system? Despite the relevance of emotional facial expressions in our daily life, to our knowledge only two studies investigated corticospinal excitability during the observation of emotional faces [33,34]. In a first study, Schutter and colleagues recorded MEPs by stimulating the left M1 at 300 ms from the presentation of pictures of happy, fearful and neutral facial expressions during passive viewing. Results showed an increase in MEP amplitudes to fearful facial expressions compared to happy and neutral expressions [33], although the study did not check whether fearful expressions elicited higher arousal in the observers. Another TMS study focusing on left M1 corticohypoglossal excitability (i.e., with MEPs recorded from the tongue), showed no consistent modulation of left M1 corticospinal excitability (i.e., with MEPs recorded from a forearm muscle) tested on a late time window (1100–1400 ms from picture presentation) during the observation of neutral facial expressions and arousal-matched happy and disgusted expressions [34]. No other studies investigated how emotional faces affect motor excitability at early vs. later time and to what extent the two different hemispheres are engaged.

Thus, the novel goal of the present work is to investigate the time course of the motor system involvement in processing emotional facial expressions and to explore the different functional modulations of M1 in the two hemispheres. MEPs to single-pulse TMS of M1 were recorded from hand muscles during presentation of pictures of happy, fearful and neutral facial expressions during an active recognition task. In two sessions, we probed corticospinal excitability of the two hemispheres by targeting both the left M1 and the right M1 during the task. To investigate both early and late modulations of corticospinal excitability [21,23], we stimulated M1 at both 150 and 300 ms after stimuli presentation. We selected visual stimuli from a validated database [35] and assessed the valence and arousal of the stimuli as well as the subjective perception of motion implied in the picture. In this way, we could check whether any differential modulation for positive and negative stimuli could be merely due to higher arousal or implied motion rather than valence.

Our study allowed us to test alternative predictions derived from the literature. First of all, it allows to clarify the facilitatory/inhibitory nature of earlier and later motor response to emotional faces. If early and later responses to emotional faces reflect enhanced motor readiness to arousing stimuli [16,17,18,19,20,21], we predicted larger MEPs at 150 ms and 300 ms for fearful and happy facial expressions and no difference between the two types of pictures, as we selected stimuli with comparable potential for arousal. Investigating MEPs at 150 ms allows to test the alternative hypothesis of reduced motor reactivity to emotional faces at this earlier timing, which could reflect early orienting/freezing response to emotional signals, as suggested by research on emotional bodies [23]. In a similar vein, if MEPs at 300 ms mainly reflect motor resonance rather than any emotion-related processes [22,23], we expected changes in motor excitability only at 150 ms and no motor modulation at 300 ms, as in the present study we recorded MEPs from the hand, not from the face, and motor resonance effects at this timing are muscle specific [36,37,38].

Notably, by targeting both the left and right M1, our design also allowed us to provide further insights on the processes underlying early MEP changes. According to the classical hypothesis of the right hemisphere dominance [39,40,41,42] we predicted larger emotion-related effects over the right M1, as the right hemisphere is more involved in processing arousing stimuli. On the other hand, according to a purely “motor” hypothesis, larger effects could be expected over the left M1, controlling the right hand, as adaptive motor reactions could in principle engage the dominant hand to a greater extent [21,23]. Although not supported by previous MEP studies, our design also allowed to test the valence-specific [40,43,44] or the motivation-specific hypothesis [43,45,46] according to which, the right M1 and left M1 would be more sensitive to fearful and happy emotions, respectively.

Finally, emotional arousing social stimuli may also trigger empathy-related processing or personal distress [21,23,47,48,49] and indeed, studies have shown that motor reactivity during social perception can be predicted by stable empathy or personal distress dispositions [21,23,26,29,50,51,52,53]. Thus, an additional novel goal of the study is to test whether interindividual differences in empathy and personal distress dispositions predict the magnitude of motor response to facial expressions.

## 2. Materials and Methods

### 2.1. Participants

Twelve healthy subjects took part in the study (6 men, mean age ± S.D.: 23.5 y ± 0.7). All participants were right-handed according to a standard handedness inventory [54], had normal or corrected-to-normal visual acuity and were free from any contraindication to TMS [55]. They gave their written informed consent to take part in the study, which was approved by the Bioethical committee at the University of Bologna and was carried out in accordance with the ethical standards of the 1964 Declaration of Helsinki. No discomfort or adverse effects during TMS were reported or noticed.

### 2.2. Visual Stimuli and Pilot Experiments

**Figure 1:**
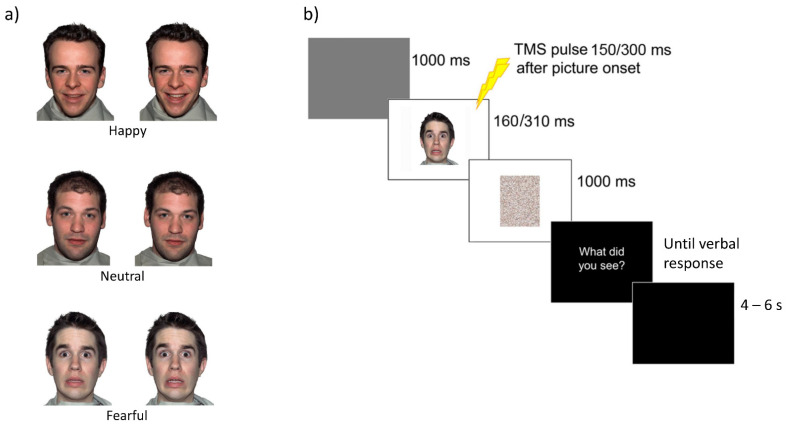
(**a**) Examples of visual body stimuli. (**b**) Trial sequence.

Visual stimuli consisted of 54 face pictures (1000 × 1500 pixels) taken from the Nimstim database [35], depicting five different male actors showing emotional facial expressions (happy and fearful) or without any expression (neutral) (Figure 1a). A total of 18 neutral, 18 fearful and 18 happy images of facial expressions were presented on a 19-inch screen located about 80 cm away from the participant.

In order to increase our pool of stimuli and to create faces showing different quantities of happiness or fear (with matched intensity and recognition accuracy), we morphed the expressions of the five actors and generated a sample of 150 stimuli using the Fantamorph software (Abrosoft, PR, Italy; https://www.fantamorph.com (accessed on 1 March 2010)). The software allowed us to morph pairs of pictures of each actor and create transitions from neutral to emotional faces. For each model, two experimenters initially selected transitions near the two extremes (e.g., neutral and happy expressions or neutral and fearful expressions), so as to start from a pool of pictures showing slightly different, but not ambiguous, facial expressions. These 150 stimuli were initially presented to a sample of 15 participants (2 males, mean age 25.1 y) who were asked to judge the intensity of happiness and fearfulness conveyed by each face using a 9 point Likert-like scale (from 1—no emotion/neutral–to 9—maximal emotional intensity). Based on the participants’ evaluation, we selected 105 stimuli where fearful and happy expressions were matched for emotional intensity. These 105 stimuli were entered in a second validation experiment, in which 24 participants (10 males, mean age 24.9 y) were requested to categorize the facial expression (3 forced choices: happy, fearful and neutral). Based on this further test, we selected the final sample of 54 pictures (18 for each expression) to be used in the TMS experiment. The selected pictures were accurately recognized with comparable percentages of correct responses (happy faces: 91%; neutral faces: 92%; fearful faces: 92%) and congruent ratings of happiness (happy faces: 5.66; neutral faces: 1.74; fearful faces: 1.17) and fear (happy faces: 1.06; neutral faces: 1.46; fearful faces: 6.21).

### 2.3. Transcranial Magnetic Stimulation and Electromyography Recording

MEPs were collected in two separate sessions testing motor excitability of the right (M1right session) and left M1 (M1left session). Both sessions started with the electrode montage, detection of optimal scalp position and measurement of resting motor threshold. To explore motor excitability, single TMS pulses were delivered over the right and left M1, and MEPs were recorded from the first dorsal interosseus (FDI) muscles, contralateral and ipsilateral to the stimulated hemisphere, with a Biopac MP-35 (BIOPAC System Inc., Goleta, CA, USA.) electromyography (EMG) system. EMG signals were band-pass filtered (30–500 Hz), sampled at 5 kHz, digitized and stored on a computer for off-line analysis. Pairs of silver-chloride surface electrodes were placed in a belly-tendon montage with ground electrodes on the wrist.

MEPs were induced using a Magstim Rapid2 stimulator (Magstim, Whitland, Dyfed, UK) connected to a figure-of-eight coil (70 mm diameter; peak magnetic field 2.2 Tesla). The intersection of the coil was placed tangentially to the scalp with the handle pointing backward and laterally at a 45° angle from the midline, to induce a posterior-anterior current flow approximately perpendicular to the line of the central sulcus. Detection of optimal scalp position and resting motor threshold (rMT) was performed as follows. Optimal scalp position was identified by using a slightly suprathreshold stimulus intensity. The coil was moved over the target hemisphere to determine the optimal position from which maximal amplitude MEPs were elicited in the contralateral FDI muscle. In the M1right and M1left experiments, the intensity of magnetic pulses was set at 125% of the rMT, which was defined as the minimal intensity of stimulator output that produces 5 MEPs with an amplitude of at least 50 μV in the relaxed muscle in a series of 10 stimuli [56]. Mean stimulation intensity (mean % of maximal stimulator output ± S.D.) were similar for M1right (71.8% ± 6.2) and M1left (70.3% ± 7.6; Wilcoxon matched pairs test: Z = 1.78, *p* = 0.09).

The absence of voluntary contraction was visually verified continuously throughout the experiments in both the left and right FDI simultaneously. When muscle tension was detected in one of the two muscles, the experiment was briefly interrupted and the subject was invited to relax.

### 2.4. Procedure and Experimental Design

The experiment was programmed using Matlab software to control picture presentation and to trigger TMS pulses. In both sessions (rightM1 and leftM1 stimulation) MEPs were collected in four blocks. The first and the last blocks served as baseline: for each block 10 MEPs were recorded with an inter-pulse interval of 10 s while subjects kept their eyes closed with the instruction to imagine watching a sunset at the beach [22,57,58]. This number of trials (*n* = 10) provides stable MEP measurement [59]. In the other two blocks, consisting of 54 trials each, subjects were presented with the face pictures and were asked to categorize them as either a happy, fearful or neutral facial expression. Each trial started with a gray screen (1 s duration), followed by the test picture projected at the center of the screen (Figure 1b). In half the trials, stimuli were presented for 160 ms and TMS was delivered at 150 ms from stimulus onset. In the remaining trails, stimuli were presented for 310 ms and TMS was delivered at 300 ms from stimulus onset. The minimal asynchrony between the TMS pulse and picture offset (i.e., 10 ms) ensured that pictures were still on the screen when M1 was stimulated. The duration of the test stimuli was randomly distributed in the blocks. For each session (right M1, leftM1), stimulus exposure (150 ms, 300 ms) and condition (happy, fearful and neutral facial expressions), we collected 18 MEPs. This number of trials provides stable MEP measurement [59] and it is well in keeping with prior TMS work on emotional faces [33,34], bodies [22,23,24,25,26,29] and scenes [16,18,19].

After the picture presentation, a random-dot mask (obtained by scrambling the corresponding sample stimulus by means of a custom-made image segmentation software) appeared for 1 s. Then the question “What did you see?” was presented on the screen, and the subject had to provide a verbal response (forced choice). Possible choices were happy, fearful or neutral. An experimenter collected the answer by pressing a computer key. To avoid changes in excitability due to the verbal response [60,61], participants were invited to answer only during the question screen, a few seconds after the TMS pulse [58]. After the response, the screen appeared black for 4–6 s, ensuring an inter-pulse interval greater than 10 s and thereby avoiding changes in motor excitability due to TMS per se [62]. This was directly confirmed by the lack of changes in FDI MEP amplitudes between the first and the last baseline blocks in both the M1right (mean ± S.D. = 1.08 mV ± 0.75 vs. 1.14 mV ± 0.46; Wilcoxon test: Z = 0.15, *p* = 0.88) and the M1left sessions (1.51 mV ± 0.89 vs. 1.63 mV ± 0.66; Wilcoxon test: Z = 1.02, *p* = 0.31). However, the two averaged baseline values differed between the two sessions, with larger MEPs in the M1left session as compared with the M1right session (1.11 mV ± 0.53 vs. 1.57 mV ± 0.75; Wilcoxon test: Z = 2.35, *p* = 0.019).

To reduce the initial transient-state increase in motor excitability, before each block two magnetic pulses were delivered over the targeted M1 (inter-pulse interval >10 s). Each block lasted about 10 min. The order of the stimulated hemisphere was counterbalanced across participants.

### 2.5. Subjective Measures

After TMS, subjects were presented with all the stimuli (shown in a randomized order) and asked to evaluate arousal, valence and perceived movement using a 10 cm visual analogue scale (VAS). Each rating was collected separately during successive presentation of the whole set of stimuli, in order to minimize artificial correlations between the different judgments. Afterwards, to assess empathy and personal distress dispositions, participants were asked to fill out the interpersonal reactivity index (IRI) [63], a 28-item self-report survey consisting in four subscales, namely perspective taking (PT, that assesses the tendency to spontaneously imagine and assume the cognitive perspective of another person), fantasy scale (FS, that assesses the tendency to project oneself into the place of fictional characters in books and movies), empathic concern (EC, that assesses the tendency to feel sympathy and compassion for others in need) and personal distress (PD, that assesses the extent to which an individual feels distress in emotionally distressing interpersonal contexts). PT and FS allow to evaluate cognitive components of empathy, while EC and PD correspond to the notions of other-oriented empathy reaction and self-oriented emotional distress, respectively [63].

### 2.6. Data Analysis

Neurophysiological and behavioral data were processed off-line. Mean MEP amplitudes in each condition were measured peak-to-peak (in mV). MEPs associated with incorrect answers were excluded from the analysis (less than 6% in both sessions). It is well established that background EMG affects motor excitability [64]; to minimize this issue, we computed the mean rectified EMG signal across a 100-ms interval preceding the TMS artifact and discarded MEPs with preceding mean EMG signal deviating from the mean of the distribution of the relevant condition by more than 2 S.D. (less than 6%). This allowed to remove motor activations that could affect MEP amplitudes [64]. In none of the participants the TMS artifact affected measurement of EMG background or MEP amplitudes. Mean accuracy in both experiments was high (right M1: mean 94.2% ± 5%; left M1: mean 95.4% ± 4.1%) and comparable across the two sessions (Wilcoxon test: Z = 0.98; *p* = 0.33).

Due to the small sample size of the study, the analysis on MEPs was carried out by nonparametric Friedman ANOVA, with Condition (right-150-happy, right-150-neutral, right-150-fearful, right-300-happy, right-300-neutral, right-300-fearful, left-150-happy, left-150-neutral, left-150-fearful, left-300-happy, left-300-neutral, left-300-fearful) as the within-subjects factor. Further Friedman ANOVAs and Wilcoxon matched pairs tests were carried out to detect the source of significant modulations. Non parametric effect size r based on Wilcoxon tests were computed following the recommendation of Rosenthal [65]. By convention, r effect sizes of 0.1, 0.3, and 0.5 are considered small, medium, and large, respectively.

Mean VAS ratings for arousal, valence and perceived movement induced by the different images were analyzed by means of three different Friedman ANOVAs with ‘type of expression’ as within-factor (happy, fearful and neutral) and Wilcoxon matched pairs tests for follow-up analyses.

Friedman ANOVA on MEPs recorded when the TMS pulse was delivered in the right M1 at 150 ms of pictures onset showed an increase in motor excitability for the faces expressing emotion compared to the neutral faces. To test whether such effect was related to individual differences in both cognitive and emotional empathy, an index of the early and lateralized motor modulation was computed (mean of effect on right M1 at 150 ms for happy and fearful expression minus the mean of effect on right M1 at 150 ms for neutral expression) and was entered as a dependent variable in different Spearman correlation analyses, whereas individual questionnaire scores from the IRI subscales were entered as predictors.

## 3. Results

### 3.1. Subjective Measures

The Friedman ANOVA carried out on valence ratings was significant (χ^2^ = 18.50, *p* < 0.001; Table 1). Wilcoxon tests showed that valence ratings were lower for fearful (2.23 ± 1.34) compared to happy (7.02 ± 1.51) and neutral (4.76 ± 0.62) facial expressions (all Z ≥ 3.06, *p* < 0.001); moreover, valence ratings were higher for happy compared to neutral expressions (Z = 2.67, *p* < 0.01).

The Friedman ANOVA on arousal ratings was significant (χ^2^ = 18.17, *p* < 0.001; Table 1); Wilcoxon tests showed higher scores for happy (5.35 ± 1.72) and fearful (6.45 ± 1.93) compared to neutral facial expressions (1.50 ± 1.08; all Z ≥ 3.06, all *p* < 0.01). Moreover, arousal ratings were not significantly different between fearful and happy expressions (Z = 1.41, *p* = 0.16).

Finally, the Friedman ANOVA conducted on implied motion ratings was also significant (χ^2^ = 18.67, *p* < 0.0001; Table 1); Wilcoxon tests showed higher scores for happy (6.37 ± 1.53) and fearful (6.72 ± 1.55) compared to neutral (1.03 ± 0.71) facial expressions (all Z ≥ 3.06, all *p* < 0.01); moreover, implied motion scores were comparable between happy and fearful expressions (Z = 1.25, *p* = 0.21).

### 3.2. Neurophysiological Data

**Figure 2:**
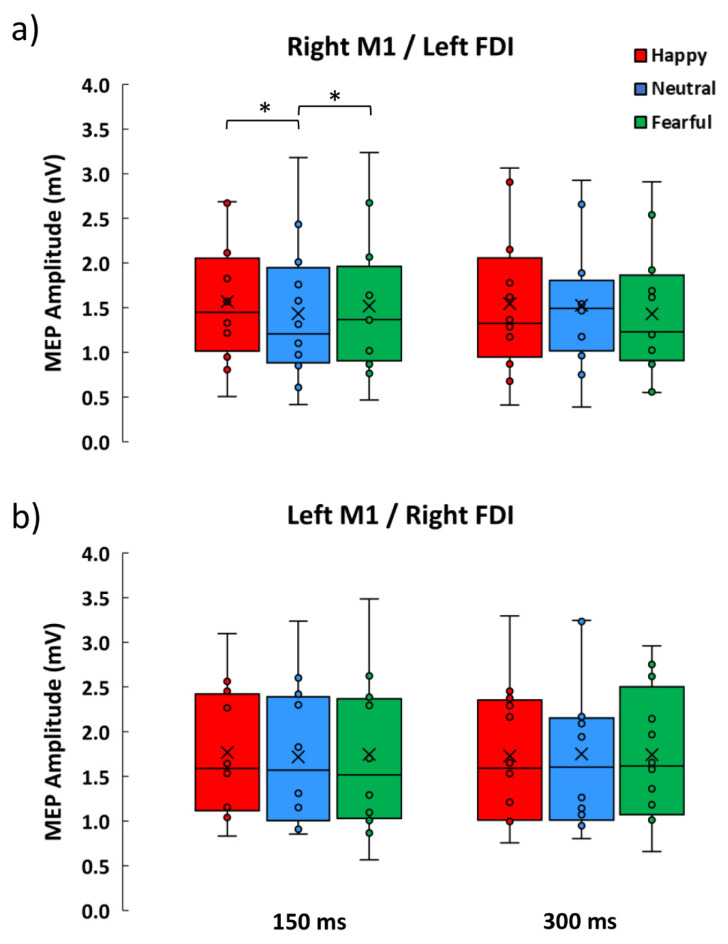
Boxplots showing MEP amplitudes recorded during the presentation of happy, neutral and fearful facial expressions at 150 and 300 ms from the stimulus onset. (**a**) Data from the right M1 experiment showing an early increase of MEPs for emotional facial expressions. (**b**) Data from the left M1 showing no MEP modulation. Asterisks denote significant Wilcoxon comparisons (*p* < 0.05).

The Friedman ANOVA conducted on MEPs amplitude was significant (χ^2^ = 21.76, *p* = 0.026; Figure 2). To explore these findings, we conducted two separate analyses, one for each stimulating session. The Friedman ANOVA on data from the M1left session was not significant (χ^2^ = 3.52, *p* = 0.62; Figure 2b), whereas the Friedman ANOVA on data from the M1right session was significant (χ^2^ = 12.43, *p* = 0.029; Figure 1a), showing consistent modulations across early time conditions (150 ms: χ^2^ = 8.67, *p* = 0.013), but not at later time conditions (300 ms; χ^2^ = 3.5, *p* = 0.17). Wilcoxon matched pairs test showed that MEPs induced by right M1 stimulation at early timing were greater in the happy (1.57 mV ± 0.69) and fearful (1.52 mV ± 0.81) conditions compared to the neutral condition (1.43 mV ± 0.80; all Z ≥ 2.04, all *p* ≤ 0.04, all effect size r ≥ 0.59); moreover, happy and fearful conditions were statistically comparable (Z = 1.02, *p* = 0.31). MEPs across the six experimental conditions of M1right sessions were larger than the corresponding baseline MEPs (all Z ≥ 2.35, all *p* ≤ 0.019, all effect size r ≥ 0.61). In contrast, no amplitude difference was observed between the active conditions of the M1left session and the corresponding baseline (all Z ≤ 2.35, all *p* ≥ 0.08).

### 3.3. Relations between Changes in Motor Excitability and Dispositional Empathy

**Figure 3:**
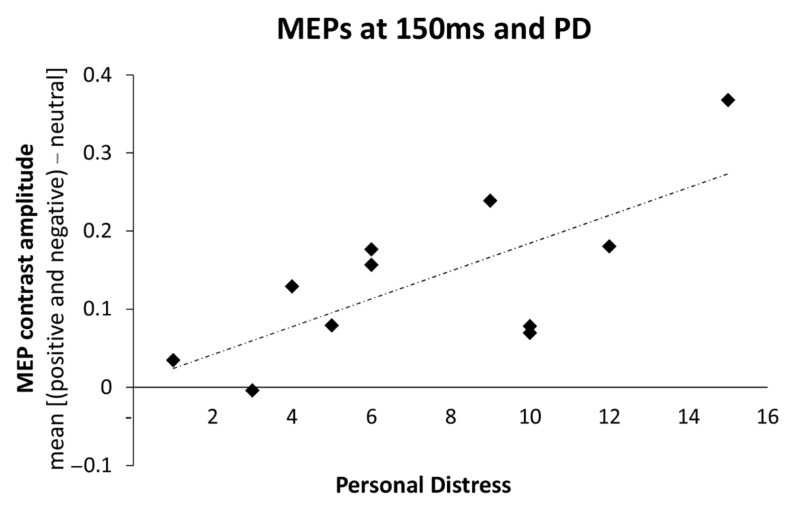
Simple correlation between MEP contrasts at 150 ms (amplitude during happy and fearful facial expressions minus neutral conditions) and the personal distress subscale of the Interpersonal Reactivity Index.

To test whether individual disposition in empathy correlates with the physiological changes induced by the processing of emotional facial expressions, Spearman’s rho correlations were used to assess the relationship between the index of early MEPs modulation for emotional faces (i.e., the increase of MEPs induced by right M1 stimulation collected in the 150 ms condition for emotional faces compared to the neutral face) and the scores on the IRI subscales. Initially, there were no significant correlations between the ratings on the IRI subscales and MEPs index (0.01 ≤ Spearman r ≤ 0.48; all *p* ≥ 0.11). However, after the removal of a statistical outlier in the data set with standard residual greater than two standard deviations, the magnitude of motor facilitation for emotional faces positively correlated with the personal distress (PD) subscale (Spearman r = 0.63; *p* = 0.037; Figure 3), suggesting that participants with higher scores in PD show greater MEPs increment for emotional faces. Other correlations remained non-significant even after the removal of the outlier (–0.02 ≤ Spearman r ≤ 0.40; all *p* ≥ 0.22).

## 4. Discussion

Decoding and rapidly reacting to emotional facial expressions represents a fundamental human ability for effective social interactions. Due to the crucial importance of reacting to emotional facial expressions, it is reasonable to expect these stimuli to affect the motor system of an observer quite early in time. However, previous TMS investigations failed to test early reactivity to emotional faces. To fill this gap, here we tested this hypothesis, by using the single-pulse TMS to monitor early (at 150 ms) and later (at 300 ms) modulations in corticospinal excitability of the right and the left M1, while participants actively categorized emotional (happy or fearful) and neutral facial expressions.

Our results show that in general, the emotion recognition task enhanced motor excitability in the right M1 compared to baseline values, whereas no similar increase was observed for the left M1, suggesting that the emotion recognition task engaged the right M1 to a greater extent than the left M1, across all conditions. Even more importantly, during the emotion recognition task, we observed an early motor facilitation for emotional faces that was selective to the right M1: when TMS was administered at 150 ms from picture presentation, left FDI MEPs during the observation of happy and fearful faces were greater than left FDI MEPs during the observation of neutral faces. No difference was found between happy and fearful face stimuli, and subjective ratings ensured that these two classes of stimuli had different valence but comparable arousal and implied motion. No similar modulations were observed at the later time (300 ms) in the left FDI or in the right FDI, indicating no changes in corticospinal excitability in these conditions. Moreover, we found that the interindividual differences in the disposition to experience aversive feelings in interpersonal emotional contexts (i.e., personal distress, as tapped by the IRI’s PD scores) predicted interindividual differences in the magnitude of early right M1 facilitation for emotional faces. These findings expand on previous research by demonstrating an early and transient facilitatory corticospinal response to emotional faces within the right M1 and showing that this response is larger in participants with greater interpersonal anxiety-related personality traits.

Our findings suggest that corticospinal excitability in the right M1 is sensitive to facial emotional expressions during an active recognition task, whereas the left M1 showed no such sensitivity. Traditionally, two main theories have linked emotion perception to the issue of hemispheric laterality. According to one view, the right hemisphere has a pivotal role in processing all emotions, whereas other views, usually known as the valence-specific hypothesis [40,43,44] or the related motivation-specific hypothesis [43,45,46], suggest that the right and the left hemispheres are relatively specialized in processing different types of emotions (i.e., negatively and positively valenced emotions, or emotion based on withdrawal or approach motivation). By demonstrating that the observation of both happy and fearful facial expressions modulates the motor excitability in the right hemisphere only, our results appear more in line with the right hemisphere dominance hypothesis than with the valence- or motivation-specific hypotheses. Moreover, we found no support for a purely motor hypothesis according to which arousing stimuli would prime the dominant (left) hand for action.

The theory of the right hemisphere dominance in the processing of emotion [39,40,41,42] was originally supported by several clinical as well as experimental findings, and it is still supported by recent evidence (for a review see [66]). However, meta-analytic neuroimaging work [5,7] indicates that both hemispheres are engaged during the processing of emotional faces and other stimuli. Emotions are the result of activations in networks which are interrelated, but may have differential lateralization patterns, and classical proposals such as the right-hemisphere dominance or valence/motivation lateralization could reflect different aspects of emotion processing [67]. A recent review highlights the possibility that the brain is right-biased in emotional and neutral face perception by default, however, task conditions can activate a more distributed and bilateral brain network [68]. In light of this, our findings may suggest that motor networks within the right hemisphere are particularly engaged during recognition of emotional faces—although we focused on M1 only and therefore it remains to be investigated how the right M1 is embedded into a wider cortico-subcortical network involved in processing emotional faces, and what its specific role is in the network.

Our findings show larger MEPs when seeing emotional faces, in keeping with prior evidence of facilitatory response to emotional stimuli using the IAPS database [16,17,18,19,20,21] or facial expression as in the study of Schutter [33]. Our findings expand on these previous works by showing that facilitatory response to emotional faces is comparable for happy and fearful expressions and can occur at early timing, that is 150 ms. Two previous works investigated MEP modulation at that specific timing. Using IAPS, Borgomaneri et al. [21] reported increased excitability for negative scenes only, although these scenes were more arousing than positive scenes. Taken together, this prior study and the present one indicates that emotional faces and emotional scenes induce an early increase in motor excitability—possibly reflecting increased readiness—and suggest that the level of arousal is a key factor in driving change in motor excitability. In a second study, MEPs from the left and right M1 were collected at 150 ms from stimulus presentation, while participants were asked to actively categorize pictures of emotional body postures [23]. In line with the present findings, both fearful and happy expressions modulated the right M1 to a similar extent (and such response was predicted by interindividual differences in the personal distress), whereas no consistent modulation was observed over the left M1. However, in contrast to the present findings, emotional body postures inhibited motor excitability, and this inhibition was interpreted as reflecting an orienting response toward emotional salient cues. Thus, while all these studies converge in showing early motor modulations, the opposite sign of the change in motor excitability remains to be accounted for. This difference may be ascribed to the different kind of stimuli, since body postures can be conceived as more complex stimuli compared to facial expressions, and may, therefore, require more resources or time to be processed (see the latency of the ERP component N170 elicited by faces versus the N190 elicited by bodies; [69,70]). In this vein, one could speculate that early inhibitory motor modulations reflecting orienting could be either specific to bodies (or to any ambiguous emotional signal) as they require more resources to be decoded. Alternatively, inhibitory modulations could also be detected for stimuli depicting facial expressions, but at an earlier timing. Future studies could directly test these alternative possibilities. Indeed, it is important to mention that here we have tested only two time points, thus it is possible that other modulatory effects may be visible at different timings. In keeping with previous research [21,22,23,24,25,26,29,33] and to limit the duration of the experiment, we focused on happy, neutral and fearful expressions. Thus, future studies should test MEPs using additional facial expressions, to understand whether early increase in motor excitability is specific to certain expressions or is a common feature of emotional face processing. Moreover, we should also consider that our sample was relatively small, and the analyses implemented were not corrected for multiple comparisons. However, the critical comparisons were associated with large effect sizes. Nevertheless, our findings warrant replication in larger cohorts, possibly testing more facial expressions and time points.

Our design allowed us to provide some insights into the late motor modulations observed in prior work using emotional scenes and bodies, specifically at 300 ms from picture presentation [21,22,23]. This previous work showed that emotional IAPS stimuli increased motor excitability compared to neutral scenes [21]. However, as discussed in the introduction, emotional scenes often show facial and body dynamic cues (i.e., motor actions), whereas IAPS neutral scenes typically depict neutral contexts with no humans, raising the possible concern that motor facilitation to emotional scenes may be due to motor resonance, rather than emotion-related modulations. On this matter, two previous studies showed that motor facilitation at 300 ms is observed not only for emotional body expressions but also neutral body movements [22,23]. Our study further supports the possibility that MEP changes at this timing might reflect motor resonance rather than emotion-related motor modulations. Indeed, there is extensive evidence that motor resonance is muscle specific [36,37,71,72], particularly around these temporal windows [38], and this can explain why facilitations of hand motor representations were observed for moving bodies [22,23], but not facial expressions (present study). In sum, our study suggests that during active emotion recognition tasks, emotional related motor modulations are likely to be observed at an early (150 ms), not later (300 ms) time. These findings motivate to further explore dynamic modulations of motor excitability using different stimulus types to disentangle the functional meaning of motor modulations. Moreover, our findings suggest future studies should also disentangle the contribution of facial expressions and dynamic bodies in complex emotional scenes.

Lastly, we found that the magnitude of early motor facilitation to emotional faces was predicted by the IRI’s PD scores. The PD scale assesses aversive, self-focused emotional reactions of personal anxiety and distress when seeing the misfortunes of others [63]. While personal distress may counteract mature forms of empathy [47,73,74], imaging studies have reported that participants who score high on the PD scale show enhanced reactivity of the insula when seeing both happy and disgusted facial expressions [75], as well as painful expressions [76], suggesting that high personal distress levels are associated with a general increase in emotional reactivity to others’ emotions. These findings are in line with electrocortical and imaging evidence showing that stronger visual cortex sensitivity to social and emotional information is linked with interpersonal anxiety-related dispositions [77,78,79]. Such a link between inter-individual differences in PD scores and the magnitude of the motor cortex reactivity was also observed in other experimental conditions, i.e., with complex negative scenes and emotional body postures [21,23], as well as during the observation of the pain of others [74], in keeping with the notion that anxiety-related traits are associated with greater motor excitability [80] and weaker motor control when facing emotional stimuli [51]. Taken together, these findings provide further support to the view that anxiety-related traits influence the way in which social and emotional signals are processed in the brain [29,53,81,82,83,84,85].

## 5. Conclusions

In conclusion, our findings provide novel evidence of an early facilitatory response to emotional faces, with grater reactivity in participants with higher personal distress. Our study highlights the importance of exploring motor system involvement in both hemispheres and with high temporal resolution, and considering interindividual differences in emotional disposition.

## Figures and Tables

**Table 1:** Mean ± S.D. of arousal, valance and implied motion ratings of the stimuli.

	Happy Expression	Fearful Expression	Neutral Expression
Valence	7.02 ± 1.51	2.23 ± 1.34	4.76 ± 0.62
Arousal	5.35 ± 1.72	6.45 ± 1.93	1.50 ± 1.08
Implied motion	6.37 ± 1.53	6.72 ± 1.55	1.03 ± 0.71

## Data Availability

The raw data that support the findings of this study are available in Open Science Framework at the following online repository https://osf.io/34cvg/?view_only=182a524ec3a84453ae17a12502461076 (accessed on 16 July 2021) or further material requests should be addressed to Sara Borgomaneri.
